# A Novel Review of the Evidence Linking Myopia and High Intelligence

**DOI:** 10.1155/2015/271746

**Published:** 2015-01-11

**Authors:** Ajai Verma, Abhishek Verma

**Affiliations:** ^1^St Vincent's Hospital, Melbourne, VIC 3065, Australia; ^2^Healthscope Private Hospitals, Woy Woy, NSW 2256, Australia

## Abstract

The association between myopia and high intelligence has been the subject of much vexed debate in academic circles, particularly over the last two decades. This debate has risen from the observation that, over recent centuries, the prevalence of myopia amongst most populations has coincided with a marked increase in the average level of intelligence in these populations. The relationship between myopia and intelligence and theories surrounding this association is examined by the authors. Additionally, the various factors that confound the myopia and high intelligence debate, such as genetics, educational levels, ethnicity, and environmental factors were also explored by the authors. Whilst most studies found a positive correlation reaching statistical significance between myopia and high intelligence compared to emmetropes and hyperopes, further research is required to determine whether this association is causal.

## 1. Introduction

Refractive defects are estimated to affect over one-third of individuals aged over 40 in the United States and Western Europe [1].* Myopia*, commonly referred to as “short-sightedness,” is a defect whereby rays of light from a distant object come to focus in front of the retina rather than on it. This is most commonly due to an enlarged axial length—the length from the posterior corneal surface to the retina—or an increase in the refractive power of the eye, usually due to a steep retina [2]. This is in contrast to* hyperopia* (also known as* hypermetropia* or “far-sightedness”), in which light is focused behind the retina due to a short eye or insufficiently curved cornea [2].

The estimated prevalence of myopia in the United States amongst 12–54-year-olds in 2004 was 42%, a figure which has nearly doubled in the prior thirty years [3]. While Caucasian Americans are the predominant race affected [4], worldwide the prevalence of myopia is particularly high amongst those of south Asian descent [5–7].

*Emmetropia*, on the other hand, is regarded as “normal refraction,” whereby parallel light rays from an object twenty feet or further form a focused image on the retina without accommodation. A person regarded as an emmetrope generally has “20/20” vision, or a visual acuity that is not deemed as requiring any corrective lenses [8].

There is a widely held perception amongst many researchers and the community that, generally, myopes tend to have superior intelligence quotients (IQs) than emmetropes. While this novel link has previously been investigated by individual studies, there is a dearth of recent literature summarising the evidence for this association. This paper aims at addressing this paucity of literature by examining the evidence for this hypothesis through a comparative analysis of the methods, intelligence testing, and results of these clinical observations. The postulated hypotheses surrounding the physiological basis underpinning this association are also examined in depth.

## 2. Defining and Measuring Intelligence

As the term* intelligence* encompasses a broad range of cognitive and psycholinguistic abilities, establishing a strict definition is somewhat difficult. Colom et al. define intelligence as a general mental ability for reasoning, problem solving, and learning [9]. He describes intelligence as integrative function that incorporates cognitive functions such as perception, attention, memory, language, or planning. For the purposes of this discussion, the definition of Rogers and Holmes, who regarded intelligence as the measurable “performance intelligence” as determined by the Wechsler Intelligence Scale, Raven Standard Progressive Matrices, or other standardised aptitude tests, will be adopted [10]. The key elements of the main aptitude tests used in the studies analysed in this review are described in Table 1.

## 3. The Association between Myopia and High Intelligence

The link between myopia and high intelligence has been independently investigated by a number of studies performed in countries as diverse as Singapore, Israel, the United States, the Czech Republic and New Zealand [11–13]. While the first study conducted on this subject was performed in 1955 by Young [14], it was not until 1958 that Nadell and Hirsch demonstrated a strong positive correlation between these two factors [15]. The results of all major studies undertaken since Young's initial study are summarised in Table 2.

### 3.1. In Children

The link between myopia and high intelligence has primarily been studied in schoolchildren aged less than 18 years. Several of these studies have been longitudinal in nature, following the progress of children at specific periods in their development and applying standardised testing in order to measure intelligence. Whilst most studies exploring this link are over two decades old, there has been a recent increase in the interest and amount of modern research examining this novel association [38].

A recent study published in Singapore evaluated the relationship between myopia and high intelligence in a group of 1204 Chinese school children aged between 10 and 12 years [34]. Intelligence was assessed using the nonverbal Raven Standard Progressive Matrix test and factors controlled included the participant's age and gender, parental myopia, father's level of education, and books read per week. This study produced similarly cogent results, with the prevalence of myopia amongst those students in the highest quartile for IQ found to be 67.9%, some 30 percentage points higher than the prevalence of myopia amongst those students in the lowest IQ quartile. Remarkably, the results of this study also showed that a statistically significant result was obtained for the odds-ratio of a child with higher intelligence also having myopia. This ratio was 2.4 (with a 95% confidence interval of 1.7–3.4). Such a result highlights that those participants with higher intelligence are between roughly two to three times more likely to have a myopic defect, compounding the notion of a correlation between these two characteristics. These results were replicated in a similar study of 994 Chinese schoolchildren undertaken by the same authors in 2007 [35].

The link between myopia and high intelligence reported amongst children in the study above appears to suggest that the association between these two characteristics is established in very early childhood. This concept is supported by the research performed by Storfer on 2,720 members of high-IQ organisations (such as Mensa), where findings illustrated that 47% of the females and 33% of the males reported very early onset myopia, that is, by the age of 10. This is compared to the “expected” 5% rate of myopia amongst age cohorts with IQ in the normal range [39]. This result indicates that any association between myopia and high intelligence would appear to involve some very early (possibly even genetic or prenatal) factor.

A number of other studies also report of myopia's coincidence with high intelligence. C. P. Benbow and R. M. Benbow, who examined a group of extremely verbally precocious junior high school children (ranked at the upper 1 in 10,000 level), found that 75% had some degree of myopia, although the range of myopia varied appreciably [31]. Similarly, Lubinski and Humphreys, in their ongoing fifty-year longitudinal study (commenced in 1971 and due for completion in 2021), found that, in every year of evaluation, students identified as exceptionally mathematically gifted also had a very high coincidence of myopic defects [40].

### 3.2. In Adolescents and Adults

Despite numerous studies exploring the possible association between myopia and raised intelligence in children, there is a paucity of literature examining this relationship in the adolescent and adult populations.

Perhaps the most illuminating study that demonstrated the ostensible link between myopia and high intelligence in young adults was that performed by Rosner and Belkin. In this study, 157,748 Israeli Jewish males aged between 17 and 19 years were assessed for their degree of refractive error as well as their performance on a standardised intelligence test. The results of this study were unequivocal: after performing a logistical regression analysis, the authors determined that there was a strongly positive statistical correlation between those participants with myopia and those with higher verbal and nonverbal intelligence scores [32]. This study presents compelling evidence that myopia and high intelligence in adolescents are indeed associated, especially with the statistical power afforded by the large sample population.

In order to quantify the relationship between myopia and high intelligence, studies have been commissioned to determine the “weight” of myopia in terms of its influence on intelligence tests. Teasdale et al. performed a study on a group of 18-year-old male Danish conscripts [33]. Two groups, one comprising of 5943 myopic men and the other having 9891 nonmyopic men, were compared for their degrees of refractive error (if present) as well as their performance on an intelligence test that included visual, verbal, spatial, logical, and numerical components. Those myopic men included in the study varied in the range of their visual acuity defect from in between −0.25 D and −7.75 D. In reviewing the results, it was found that the myopic men correlated with superior intelligence test scores (a positive correlation coefficient of 0.572, at the significance level of *P* < 0.001) than did their emmetropic counterparts. By analysing the data collected in the study and employing advanced statistical methods such as the Scheffe analysis of variance test, the authors concluded that having myopia yielded, on average, seven IQ points to myopic men over their emmetropic counterparts.

## 4. The Scientific Basis behind the Link between Myopia and High Intelligence

Much scientific conjecture exists as to how and why myopia and high intelligence might be associated. As early as 1959, Hirsch propounded several hypotheses regarding this link [27]:

1. myopia which represents overdevelopment of the eye, with ocular and cerebral development being related;
2. the amount of reading done by a child influences their intelligence scores; myopic children, who are “better adapted” to reading than their hyperopic counterparts, therefore score better on intelligence testing;
3. intelligence, as opposed to refraction, which determines the amount of reading that a child does. More intelligent children have a higher likelihood of becoming myopic secondarily due to increased reading rates;
4. myopes which require less accommodation than hyperopes; therefore, they have an advantage in perceiving fine detail during testing than their counterparts due to the attendant difficulty in maintaining accommodation.

Hirsch strongly espoused the fourth hypothesis, especially in view of his data which supported this assertion. Whilst future researchers, such as Young [28], have largely been critical of his supposition of a relationship between refraction and intelligence, the credibility of environmental factors governing the association between myopes and superior intelligence has been acknowledged in other studies [29].

In a more recent study comparing children who were both myopic and highly intelligent with their emmetropic and less intelligent siblings, Cohn et al. suggested that a pleiotropic relationship between myopia and high intelligence may exist, whereby a single gene in the human genome might be responsible for controlling both characteristics [41]. This hypothesis was supported by Karlsson [30, 42] and elaborated by Mak et al., whose thesis was based on the concept that myopia, effectively being of impaired of long-distance vision, would be a trait selected against an evolutionary model, due the disadvantage it confers for the previously hunter-gatherer lifestyle of humans [43]. However, this notion is discordant with the increased rates of myopia that have been observed in almost all populations, especially in recent times. Hence, Mak postulated that intelligence and myopia might be related by a single pleiotropic genotype (nominally called* EBG*: the “Eye-Brain Gene”), which gives rise to two distinctive yet related phenotypes, namely,* (a)* neurocognitive development yielding superior intelligence and* (b)* myopia. According to Mak's theory, the myopia trait* (b)* remained latent and would not be expressed unless precipitated by some novel environmental factor, while the superior intelligence trait* (a)* leads to the strong selection for* EBG*, as superior intelligence allowed humans to refine their hunting, farming, and foraging techniques. The myopia component* (b)* of* EBG* was of little detriment as it was not manifested in the ancestral environment of humans and, henceforth, was selectively neutral. As a result, Mak suggested that there was a net gain in Darwinian fitness and* EBG* attained a very high gene frequency in the human population. However, when the population with the* EBG* genotype was exposed to certain environmental factors, for instance, large amounts of intense near-work, then the phenotype* (b)* myopia would be expressed. Thus, in modern times where there is significant near-work activity such as large volumes of schoolwork, studying, television, and video game activity, myopia becomes a much more commonly expressed trait [43]. Based on these hypotheses and postulations, Mak sought to explain the high coincidence of myopia and high intelligence that has been so widely reported.

While the theory proposed by Mak is plausible, albeit contentious, others have also suggested that genetic factors might explain the association between myopia and high intelligence. Miller proposed that since some parts of the eye and the brain have similar origins in embryology (neural ectoderm), a single gene coding for a single protein might produce some factor that affects the growth of both the brain and the eyeball [44]. This theory was based on H. von Moers-Messmer's 1940 assertion that the intelligence-myopia correlation was “ontogenetic wherein the overdeveloped eye is part of the overdeveloped brain.” This claim was founded on the observation that myopic people, who tended to exhibit “intelligence beyond the norm,” were noted to have “enlarged eyes, in particular, an increased axial length dimension.” Accordingly, Miller suggested that if large brains lead to high intelligence and large eyes lead to myopia, some factor might be accountable for the increasing the size of both of these organs, leading to the coincidence of myopia and high intelligence. While some doubt has been cast on Miller's theory by subsequent MRI studies that have analysed brain and eye size, refractive error, and intelligence, the notion that brain and eye size might influence the myopia-intelligence relationship has not been entirely discarded [44].

Storfer proposed a multifaceted argument of how myopia might be related to high intelligence [39]. He suggested that the human neocortex underwent evolutionary enlargement under the influence of environmental strains, whereby modern visual inputs, which have become increasingly variable and complex, stimulate the cortical visual and association areas of the brain and force them to expand. Furthermore, afferent and reciprocal neuronal networks in the visual pathway also enlarge in order to accommodate the increasingly complex modern visual stimuli. By extension, Storfer then hypothesised that these cortical changes allowed an opportunity for further neurocognitive development and superior intelligence, a trait heritable via genomic imprinting. In relating this brain expansion and intelligence to myopia, Storfer speculated about the existence of a biochemical mediator between the eyes and the central visual pathway whereby enlargement of the visual pathway provides some impetus that increases the ocular length and axial length of the eye and thereby causes myopia [39]. This complex hypothesis as to how myopia and high intelligence are related has been met with only lukewarm approval by other researchers. In his recent commentary (2000), Miller was particularly critical of Storfer's reticence to attribute his observations to genetic mechanisms in a commentary [45]. He refers to Curtin's work [2] when refuting Storfer's assertion regarding a “new, brain-centred theory” and also points out that high levels of reading and near-work generally correlate with increased levels of myopia and intelligence [46, 47]. However, he qualifies this statement by pointing out the high heritability of myopia amongst parents and children [48].

While the mechanism of the association between myopia and high intelligence remains controversial, the literature findings discussed above represent only a few of a number of studies that have reported on the validity of a significant link existing between myopia and high intelligence. The fact that these studies, carried out in different decades and different countries, have consistently produced strong favourable evidence to support a correlation between myopia and intelligence lends credibility to the link between these two characteristics. However, the testability of these assertions remains debatable and further research using functional imaging and genotype testing is required in order to substantiate these assertions.

## 5. Factors and Study Limitations Confounding the Myopia-High Intelligence Link

Whilst there is a strong body of evidence to support an association between myopia and high intelligence, there are also a range of factors that confound the link between these two phenomena which merit consideration.

The association between myopia and high intelligence is clouded by arguments such as those raised by Mutti et al., who assert that the link between these two phenomena may simply be an artefact of behaviour [8]. This argument centres on the fact that children who have the behavioural habit of reading more or engaging in other similar intellectual activities would naturally have a higher performance on intelligence tests, while at the same time, have a greater disposition to myopia since they are engaged in a large amount of near-work such as reading. Thereby, Mutti suggests that in this way, myopia and high intelligence may be coincident as a result of behaviour and not actually associated biologically.

An extension of this argument is that while myopia and high intelligence may be coincident, their association may be explained by environmental or heredity factors rather than any real biological association. Some researchers have observed that those children who tend to have higher intelligence and have myopia are both more likely to have (a) one or more myopic parents and (b) parents who encourage reading and intellectual activities, thereby providing a “myopigenic environment” that contributes to intelligence and fosters myopia [8]. To substantiate this thesis, Mutti conducted a study on a group of 366 school children, comparing the myopes and the emmetropes for the time per week engaged in near-work activities such as studying and reading, the number of parents they had with myopia, and their performance on a standardised intelligence aptitude test. It was found that statistically significant correlations (at the *P* < 0.0001 level) existed between the time children spent studying, their refractive error, and their performance on the intelligence test. Similarly, it was found that those children who had myopia and demonstrated a superior performance on the test were 3.32 times more likely to have one myopic parent and 6.40 times to have two myopic parents [8]. Mutti then speculated that these myopic parents, who may or may not have superior intelligence themselves, would have encouraged an environment involving significant near-work activity for their children, thereby leading to the refractive errors and higher intelligence trends observed. This study, which demonstrates the influence of a number of factors on the myopia-high intelligence, reflects the difficulty in delineating a conclusive relationship.

An important consideration in determining the validity of a myopia-high intelligence association is the factor of ethnicity. As cited by Miller, intelligence and myopia do appear to covary amongst ethnic groups, thus yielding a risk that correlations observed within populations reflect primarily ethnic effects [44]. While discussing the intelligence of different ethnic groups may be highly controversial, some authors have observed that higher intelligence test scores tend to be recorded amongst the Chinese, Japanese, and Jews—who have high incidences of myopia—than other races such as Gabon Negroes and Eskimos, who have much lower rates of myopia [49]. While there may be many reasons for this discrepancy and it is imprudent to suggest that actual differences in intelligence levels exist amongst races, at the very least, this observation does cast a question-mark over how real the association is between myopia and high intelligence in the general population, as compared to an ethnically stratified subpopulation basis. Indeed, further elaboration on this thesis has been performed, including a recent Australian study by Ip et al. In this study (the Sydney Myopia Study), 2533 children underwent ophthalmic examination, and their degree of refractive error was compared with their ethnicity and the amount of near-work they reported [49]. The findings demonstrated that myopia did indeed vary amongst the ethnicity groups and was more prevalent in children of East Asian ethnicity than those from European Caucasian backgrounds. Given the ethnic variation in incidence of myopia, the potential confounding effect of this link further complicates the myopia-high intelligence association.

Other schools of thought seek to dispel the validity of the myopia-high intelligence link purport that myopia is entirely environmental whereas intelligence has a genetic basis. In support of such theories, researchers have conducted trials in monkeys, where myopia has been artificially induced by suturing eye-lids closed or inserting distorting lenses. These myopic monkeys were compared to age-matched emmetropic cohorts for their intelligence levels, which was measured by their ability to perform certain complex spatial and orientation tasks. It was found that no statistically appreciable difference in measured intelligence was recorded between the two groups [44], thus suggesting that the myopia and high intelligence may be more related to environment than any other factor.

Not all studies investigating the potential link between myopia and high intelligence have demonstrated a significantly positive correlation between these two factors. In their study of 137 Iranian schoolchildren aged between 10 and 14 years, Akrami et al. found no statistically significant difference in the IQ and test scores between children with myopia and those with other or no refractive errors [37]. Similarly, Dirani et al. also found no significant association between myopia and intelligence test scores in a study of 1143 Singaporean schoolchildren aged 9-10 years [36]. Additionally, Ong et al. found in recent Singaporean study of 1032 patients aged 60–79 years that those with refractive errors (both corrected and uncorrected), including myopia and hyperopia, were significantly more likely to have cognitive dysfunction after correcting for demographic and educational factors [50].

Clearly, a number of issues complicate the myopia-high intelligence debate and whether a real association or artificial association exists between these phenomena remains to be conclusively established. However, despite the unproven nature of the mechanism of such a link, the bulk of current literature presents cogent evidence that myopia and high intelligence may indeed be significantly associated. Therefore, it is reasonable to suppose that further studies—especially proposed novel trials involving blind and illiterate populations—are likely to yield greater concordance in results as well as reconcile a number of the confounding factors.

## 6. Conclusion

The studies analysed in this review suggest that there may be a positive association between myopia and high intelligence. While the mechanism of the link between these two phenomena is not clearly understood and is confounded by a number of factors, there is evidence to suggest that both environmental and genetic factors may contribute to this relationship. In view of the data presented by independent and replicated studies in different countries, further research—particularly in older populations—to establish the veracity of this association is encouraged.

## Figures and Tables

**Table 1:** Description of key elements and validity of main intelligence tests.

Test	Developer	Format	Age	Areas tested	Scientific reliability and validity
Raven Progressive Matrix Test [16]	John C. Raven	Nonverbal 60 multiple choice questions	5–elderly	General intelligence educational ability	Demonstrates good convergent validity and slightly impaired discriminant validity [17, 18]

Otis Test [19]	Arthur Simon Otis	Multiple choice—verbal and non-verbal areas tested 21 subtests organised into five areas (comprehension, verbal reasoning, pictorial reasoning, quantitative reasoning, figural reasoning)	Pre-kinder–18	Verbal, quantitative, and spatial reasoning ability	Easy to administer but some concerns about less reliable accuracy at higher levels [19]

Stanford-Binet IQ Test [20]	Alfred Binet Theodore Simon	Verbal and nonverbal subtests Tests five different areas	2–85+	Knowledge Quantitative reasoning Visual-spatial reasoning Memory Fluid reasoning	Substantial split-life reliability compared to other tests [21] High precision at advanced levels of testing [21] High level of construct and comparative validity [21]

California Test of Mental Maturity [22]	Elizabeth Sullivan Willis Clark Ernest Tiegs	253 items in 16 subtests over 2 × 45 minute periods	5+	Logical reasoning Spatial ability Verbal ability Numerical ability Memory	Some concerns about difficulty of items and validity in extrapolating results to predict school performance [23, 24] Outdated test

Wechsler Intelligence Scale for Children [25]	David Wechsler	Verbal test taking 48–65 minutes	6–15	Verbal comprehension Visual spatial Fluid reasoning Working memory Speed processing	High levels of convergent, construct, and discriminant validity [26]

**Table 2:** Summary of study results linking myopia and high intelligence.

Study	Country	Number of subjects	Age (years)	Range of myopia	Intelligence test performed	IQ	Test/school results	Significance level^∗^
Young 1955 [14]	USA	633	6–17	Not specified	Stanford-Binet	Average	N/A	*P* < 0.001 CC^∗∗^ = −0.19

Nadell and Hirsch 1958 [15]	USA	414	14–18	Not specified	CTMM	Higher	N/A	*P* > 0.1

Hirsch 1959 [27]	USA	554	6–17	Not specified	Stanford-Binet CTMM	Higher	N/A	*P* < 0.001

Young 1963 [28]	USA	251	5–17	Not specified	Stanford-Binet CTMM	Average	Higher	CC = −0.11/0.10 CC = −0.17/0.21

Grosvenor 1970 [29]	New Zealand	707	11–13	≥−1.00 D	Otis Test	Higher	Higher	*P* > 0.05 (versus emmetropes) *P* < 0.05 (versus hypertropes)

Karlsson 1976 [30]	USA	2527	17-18	Not specified	Lorge-Thorndike IQ Test CTMM	Higher	Higher	*P* < 0.001

C. P. Benbow and R. M. Benbow 1984 [31]	USA	416	13	Not specified	Scholastic Aptitude Test	Higher	Higher	*P* < 0.05

Rosner and Belkin 1987 [32]	Israel	157 748	17–19	≤6/7.5 VA	Verbal Otis Test, Raven Matrix Test	Higher	Higher	Strongly positive association

Williams et al., 1988 [13]	New Zealand	537	7–11	≥−0.5 D	WISC-R IQ Test Burt Word Reading Test	Higher	Higher	*P* < 0.05

Teasdale et al., 1988 [33]	Denmark	15 834	18	≥−0.25 D to ≤−7.5 D	Group IQ Scores Educational levels	Higher	Higher	*P* < 0.001

Dolezalova and Mottlova 1995 [12]	Czech Republic	225	14–18	Unknown^†^	School test scores	Higher	Higher	Unknown^†^

Saw et al., 2004 [34]	Singapore	1204	10–12	≥−0.5 D	Raven Matrix Test	Higher	N/A	*P* < 0.05

Saw et al., 2006 [35]	Singapore	994	7–9	≥−0.5 D	Raven Matrix Test	Higher	Higher	*P* < 0.05

Dirani et al., 2010 [36]	Singapore	1143	9-10	≥−0.5 D	Raven Matrix Test	Average	Average	*P* > 0.38 (9-year-olds) *P* > 0.27 (10-year-olds)

Akrami et al., 2012 [37]	Iran	137	10–14	≥−0.5 D	Unspecified school tests	Average	N/A	*P* = 0.465

^*^ *P* score < 0.05 denotes statistically significant result.

^∗∗^CC: correlation coefficient.

^†^Data unavailable in English language at time of the literature review.
